# Fabrication of Micro-Needle Electrodes for Bio-Signal Recording by a Magnetization-Induced Self-Assembly Method

**DOI:** 10.3390/s16091533

**Published:** 2016-09-20

**Authors:** Keyun Chen, Lei Ren, Zhipeng Chen, Chengfeng Pan, Wei Zhou, Lelun Jiang

**Affiliations:** 1 Guangdong Provincial Key Laboratory of Sensor Technology and Biomedical Instrument, Sun Yat-Sen University, Guangzhou 510006, China; yunzibetterlife@163.com (K.C.); rlei@mail2.sysu.edu.cn (L.R.); asm0844@163.com (Z.C.); panchf@mail2.sysu.edu.cn (C.P.); 2 Department of Mechanical and Electrical Engineering, Xiamen University, Xiamen 361005, China; weizhou@xmu.edu.cn

**Keywords:** micro-needle array, electrode, magnetization-induced self-assembling, insertion, impedance, EMG, ECG

## Abstract

Micro-needle electrodes (MEs) have attracted more and more attention for monitoring physiological electrical signals, including electrode-skin interface impedance (EII), electromyography (EMG) and electrocardiography (ECG) recording. A magnetization-induced self-assembling method (MSM) was developed to fabricate a microneedle array (MA). A MA coated with Ti/Au film was assembled as a ME. The fracture and insertion properties of ME were tested by experiments. The bio-signal recording performance of the ME was measured and compared with a typical commercial wet electrode (Ag/AgCl electrode). The results show that the MA self-assembled from the magnetic droplet array under the sum of gravitational surface tension and magnetic potential energies. The ME had good toughness and could easily pierce rabbit skin without being broken or buckling. When the compression force applied on the ME was larger than 2 N, ME could stably record EII, which was a lower value than that measured by Ag/AgCl electrodes. EMG signals collected by ME varied along with the contraction of biceps brachii muscle. ME could record static ECG signals with a larger amplitude and dynamic ECG signals with more distinguishable features in comparison with a Ag/AgCl electrode, therefore, ME is an alternative electrode for bio-signal monitoring in some specific situations.

## 1. Introduction

**Figure 1:**
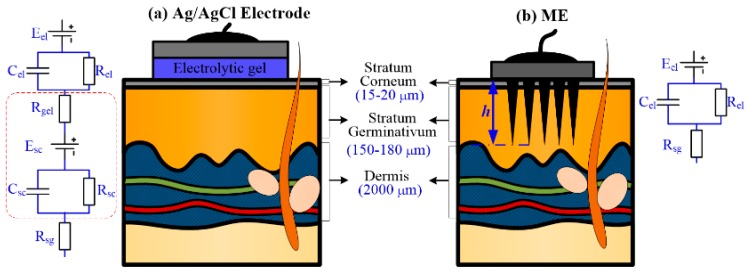
Interface between skin and: (**a**) wet electrode; and (**b**) ME.

**Figure 2:**
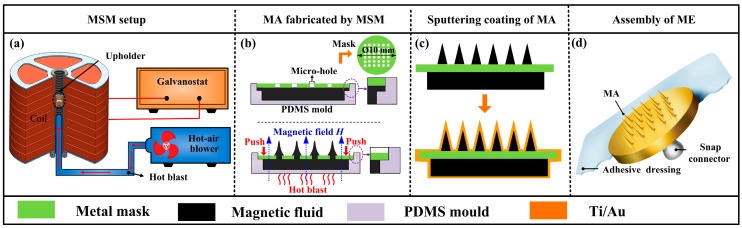
ME fabrication process: (**a**) The magnetization-induced MA equipment; (**b**) MA formation by MSM; (**c**) Sputtering coating Ti/Au films on the surface of MA; and (**d**) rendered image of ME.

Bio-signals, such as electrode-skin interface impedance (EII), electromyography (EMG), electrocardiography (ECG) and so on, are important physiological parameters and widely used in medical treatment, disease monitoring and medical research. Bio-signals are difficult to obtain due to their weak and unstable characteristics [1,2]. Electrodes are good biosensors used to collect these bio-signals [3]. Wet electrodes, dry electrodes and micro-needles electrodes (MEs) are typical ones used for bio-signal monitoring. Wet electrodes, such as typical commercial Ag/AgCl electrodes, are the most popular non-invasive electrode in clinical practice. However, several drawbacks limit their further application: (1) wet electrodes cannot reach the conductive layers of the epidermis due to the stratum corneum which hinders the extraction of signals, as shown in Figure 1a; (2) the necessary conductive gel may cause skin irritations and allergic reactions; (3) wet electrodes are not suitable for long-term bio-signal monitoring due to the slow drying of the conductive gel and increase of EII [4,5,6,7]. To eliminate the need for gel application dry electrodes are employed [8,9], however, the stratum corneum must be removed to decrease EII before bio-signal recording. This skin preparation process may damage or irritate the skin. Furthermore, dry electrodes is so sensitive to human motion that the signals may be easily disturbed. MEs are proposed to solve these problems. A ME has the following advantages: (1) it can puncture through the stratum corneum and eliminate the influence of the stratum corneum on the impedance, as shown in Figure 1b. The puncture process is almost painless; (2) the contact interface between the ME and the skin is more stable and thus can attenuate motion artifact; (3) the possibility of skin irritation or allergies can be eliminated without skin preparation [5,6,10]. However, MEs also have the disadvantage that the microneedles are easily broken during the insertion process [5,11]. This is a big challenge for practical ME applications.

A lot of researchers have reported the fabrication and bio-signal recording performance of MEs. Silicon, metal and polymer were always adopted to fabricate MEs. Photolithography technology with wet or dry etching has been widely used in the fabrication of MEs from silicon wafers. O’Mahony et al. [6] fabricated an ultra-sharp silicon ME by double-sided silicon wafer patterning and anisotropic potassium hydroxide wet etching. A ME was simultaneously created on the front side of the wafer and a through-silicon via from the backside. The ME could accurately sense the ECG and EMG with good fidelity. Yu et al. [12] proposed hollow MEs through twice deep reactive ion etching and this ME could acquire ECG signals with a high signal to noise ratio. Hsu et al. [13] fabricated barbed micro tip-based electrode arrays by silicon wet etching. The contact impedance of the barbed dry electrode was low, and ECG recordings had adequate quality. Wang et al. [14] fabricated a pyramidal silicon ME by cutting, isotropic wet-etching and sputtering. The ME showed good performance for long-term EEG measurements. Wang et al. [15,16] developed a flexible silicon micro-needle electrode array by etching and parylene deposition technology. The flexible micro-needle electrode array was promising in invasive neural electrical stimulation and recording, however, silicon needles may break off during the insertion process and stay in the skin due to their mechanical fragility. Besides, the use of lithography and etching technology to produce a disposable device is relatively expensive and complicated [17,18]. Therefore, metals were adopted to fabricate MEs because of their good strength. Liu et al. [19] fabricated a 3D ME by a micro-molding technology with liquid metal at room temperature. The ME could acquire electrical signals with great electric characteristics. Zhou et al. [10] employed a nanosecond laser micromachining process to fabricate microstructure arrays on the surface of a bio-electrode. The results showed that a copper dry bio-electrode had less contact impedance value and better stability than a Ag/AgCl electrode. Nanosecond laser micromachining is a simple method to fabricate MEa, but the machined surface of the micro needles was rough and the tips were relatively blunt. The biocompatibility of pure copper also needs to be further discussed. Some polymers have good enough rigidity and flexibility for skin insertion of MEs. Wang et al. [20] fabricated a 3D ME by a parylene-based shape-transferring technique. The ME impedance was about 72.7% of the planar one at 1 kHz in vitro, and 71.5% in vivo. Nishinaka et al. [21] fabricated a polymer ME by photolithography with micro-molding and vapor deposition technology. The ME served as a flexible electrode for a brain-machine interface. Ren et al. [22] fabricated a ME using PLGA by a thermal drawing method and coated it with Ti/Au film for bio-signal monitoring. The results showed that the ME had good biocompatibility and could record EMG, ECG and EEG signals with good fidelity in shape and amplitude in comparison to commercial Ag/AgCl electrodes. Therefore, MEs are promising alternative electrode in some specific bio-signal recording situations.

A novel magnetization-induced self-assembling method (MSM) was proposed to fabricate MA in this paper. The fabrication method is simple and low in cost. A MA coated with Ti/Au was assembled as the ME. This ME has good strength for skin insertion. In the following work, we will discuss the mechanism of MSM for the formation of MA and present the ME fabrication process. The ME structure will be characterized. Mechanical properties and bio-signal recording performance of ME will also be investigated.

## 2. Materials and Methods

### 2.1. ME Fabrication

The ME fabrication process is shown in Figure 2.

#### 2.1.1. Material Preparation

Epoxy Novolac resin was mixed uniformly with iron powder. The mass ratio of epoxy Novolac resin to iron powder was 1:5. Curing agent (modified aliphatic amine) was mixed with the mixture prepared in the first step as the magnetic fluid. The volume ratio of curing agent to the mixture was 1:2.5. The physical properties, chemical formulas and polycondensation equation of epoxy novolac resin and modified aliphatic amine are given in Table 1. Iron powder was chosen as magnetic powder due to its high magnetization intensity and its parameters are given in Table 2. The circular 316L stainless steel mask (diameter of 10 mm, thickness of 0.3 mm) was cut with 36 micro-hole array (micro-hole diameter of 0.17 mm) by a pulsed fiber laser carving machine (IPG, No.: YLP-1-100-20-20-CN, Burbach, Germany).

#### 2.1.2. Micro-Needle Array (MA) Fabrication

MSM was developed to fabricate MA. The magnetization-induced MA equipment is shown in Figure 2a. This equipment consisted of a uniform magnetic field generator and a hot-air blower. The magnetic field generator was composed of 10 set copper coils, a high power galvanostat, and an upholder. It could generate an adjustable uniform magnetic field. The hot-air blower was employed to heat and solidify the MA. Magnetic fluid was poured into a polydimethylsiloxane (PDMS, Dow Corning, Midland, MI, USA) mould and the perforated mask was assembled on the PDMS mould as shown in Figure 2b. The abovementioned assembly was placed on the upholder in the magnetization-induced MA equipment. The equipment was turned on with a magnetic field intensity of 0.5 T. The perforated mask was pressed downwards to the magnetic fluid with a constant force until the mask touched the mould as shown in Figure 2b. The magnetic fluid was extruded from the micro-holes of mask and formed the droplet array, which was magnetization-induced and formed into the MA structure as shown in Figure 2b. MA was heated and solidified using a hot air blower at about 75 °C for 3 h as shown in Table 1. The solidified MA was lifted off from the PDMS mould. For more details about the parameters of MSM, please consult our previous paper [23].

#### 2.1.3. MA Coating

10 nm Ti film and 100 nm Au film were uniformly coated on the whole surface of MA in sequence by the magnetron sputtering machine (MSP-3300, Beijing Jinshengweina Technology Co., Ltd., Beijing, China) as shown in Figure 2c. MA coated with Ti/Au films was observed by scanning electron microscopy (SEM, JSM-6380LA, JEOL, Tokyo, Japan).

#### 2.1.4. ME Assembly

A standard-shape snap connector was soldered at the back of the MA. Medical adhesive dressing was bonded on the back of snap connector. The assembly of the ME is shown in Figure 2d.

### 2.2. Mechanical Tests

#### 2.2.1. Fracture Test

**Figure 3:**
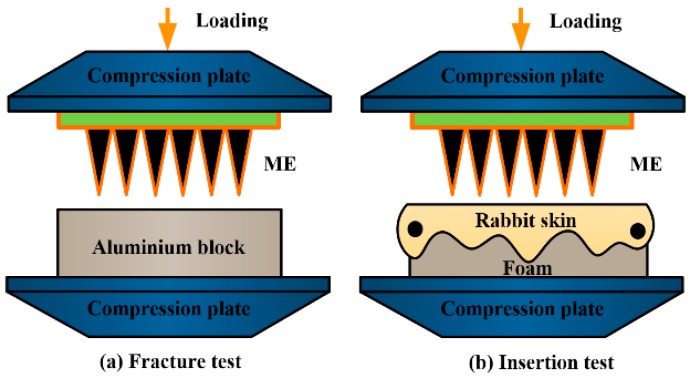
Schematic illustration of (**a**) fracture; and (**b**) insertion test ex vivo.

ME has the disadvantage that microneedles may be fractured in the skin during the insertion process and cause skin infections. Robustness determines whether the ME can puncture the skin without being broken. The fracture test was performed on a universal test machine (LR10K Plus, Lloyd Instruments, Bognor Regis, UK). The range of the force sensor is 100 N. The fracture test procedures were performed as follows: (1) ME was fixed on the upper compression plate as shown in Figure 3a. An aluminum block (size: 5 cm × 5 cm × 2 cm) was fixed on the bottom compression plate; (2) The ME was moved downwards the aluminum block at a speed of 0.01 mm/s. The loading force and displacement were recorded simultaneously; (3) The test was stopped when the loading force was 45 N; (4) The ME test results were analyzed using SEM (JSM-6380LA, JEOL).

#### 2.2.2. Insertion Test ex Vivo

ME should penetrate through the stratum corneum layer and eliminate the impedance of the dead skin to collect bio-signals [24]. Therefore, the insertion process of ME needs be further studied. The schematic illustration of insertion test ex vivo is shown in Figure 3b. The insertion test procedures were as follows: (1) micro-needle tips of ME were dipped with rhodamine B solution and dried in air; (2) ME was fixed on the upper compression plate of universal test machine (LR10K Plus, Lloyd Instruments, Bognor Regis, UK); (3) Hairless skin of a male New Zealand rabbit (2–3 months, 3.0 kg) was cleaned and cut into squares in size of 3 cm × 3 cm. Its thickness was about 1.1 mm; (4) Square skin was pre-tensed and fixed on the polymer foam which mimicked the soft tissue under the skin [25]; (5) ME was loaded and inserted into skin at a speed of 0.01 mm/s. The loading displacement and force were recorded simultaneously; (6) Test was stopped and unloaded when the loading force reached 10 N; (7) The punctured rabbit skin and ME were observed by an inverted fluorescence microscope (ECLIPSE Ti, Nikon, Tokyo, Japan) and SEM (JSM-6380LA, JEOL), respectively.

### 2.3. Bio-Signals Recording

To better understand the bio-signal recording performance of ME, EII, EMG and ECG recording tests were performed with both the self-fabricated ME and commercial Ag/AgCl electrodes (JK-1(A~H) type, Shanghai Junkang Medical Supplies Ltd., Co., Shanghai, China). The Ag/AgCl electrode, a typical wet electrode, has been widely applied in bio-signal monitoring. The measurement was firstly performed with the ME and then repeated with Ag/AgCl electrodes under the same test conditions. The experiments were tested on three healthy volunteers aged from 23 to 26 at the room temperature and repeated at least five times. The study was approved by the ethics committee of the Work Injury Rehabilitation Center of Guangdong Province (approval number: AF/SC-07/2016.29). All volunteers provided written informed consent.

#### 2.3.1. EII Test during the Insertion Process

**Figure 4:**
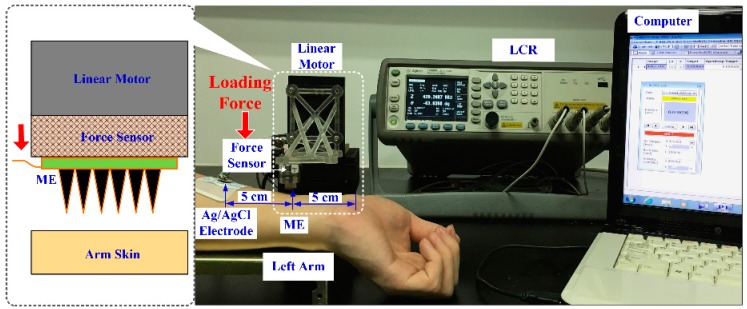
Setup designed for EII recording during the insertion process.

A setup designed for the EII recording during the ME insertion process is shown in Figure 4. A two-electrode measurement method was employed to record EII [3,22]. The placements of the ME and Ag/AgCl electrode are shown in Figure 4. The electrodes were connected to a precision impedance analyzer (E4980A LCR Meter, Agilent, Palo Alto, CA, USA). A linear motor (E-861, PI, Karlsruhe, Germany) with a force sensor (Nano 17 Titanium, ATI Industrial Automation, Detroit, MI, USA) were used to load the ME on the forearm. The EII signal, insertion force, and insertion displacement could be simultaneously recorded by a self-developed software. 

In order to better understand the effect of insertion force on EII recording, the EII recording properties of the ME during the insertion process were tested. The left inner forearm was chosen as the measurement site due to its features of less hair and thinner stratum corneum [10,26]. The speed of the linear motor was 0.5 mm/s; the injection voltage of the LCR meter was 1 V; and its frequency was 50 Hz. We also recorded EII with input voltage frequency ranging from 20 Hz to 10 kHz under a constant compression force (about 1, 2 or 3 N). If the impedance measured was greater than 2 MΩ, the testing result would be set as 2 MΩ.

#### 2.3.2. EMG Test

**Figure 5:**
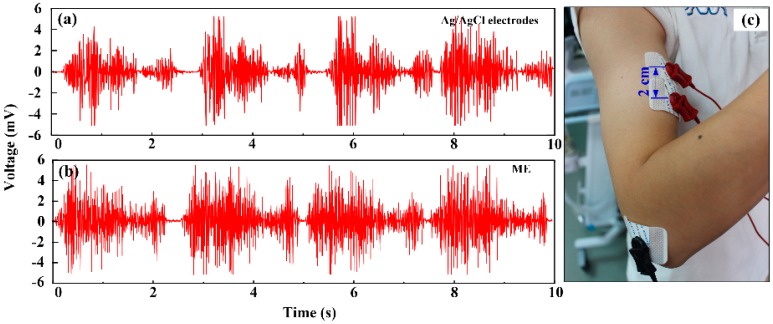
EMG recorded by: (**a**) Ag/AgCl electrodes; and (**b**) ME; (**c**) Recording positions.

The biceps brachii muscle was chosen as the EMG monitoring object and a differential method was employed to record EMG signals. The placement positions of the electrodes are shown in Figure 5c. Two measuring electrodes (such as ME or Ag/AgCl electrodes) were placed on the biceps brachii muscle with an interval of 2 cm. The grounding electrode (Ag/AgCl electrode) was on the elbow. The electrodes were connected to a tele-EMG system (MyoSystem2400T, Noraxon, Scottsdale, AZ, USA). The volunteers held a 1.5 kg dumbbell and flexed the elbow every 2–3 s and EMG signals were recorded. The test would last about 5 min. During the test, the amplifier gain was set at 2000, and the sample rate was set at 1000 Hz. A 20 Hz–450 Hz Butterworth band-pass filter was used.

#### 2.3.3. ECG Test

ECG signals in the static and dynamic state were collected by the ME to evaluate its performance in comparison with the Ag/AgCl electrode. The ECG was recorded by a standard II-lead method. Measuring electrodes (ME or Ag/AgCl electrodes) were stuck on both wrists and grounding electrode (Ag/AgCl electrode) was on the right ankle. Three electrodes were connected to ECG100C module of a Multipurpose Polygraph (MP150, BIOPAC, Goleta, CA, USA). The volunteers lay on a bed during the static ECG recording while walking on a treadmill at a uniform speed of 3 km/h during the dynamic ECG recording. The test lasted at least half an hour. During the test, the amplifier gain was set at 5000, the sample rate was set at 1000 Hz, the high-pass filter was set at 0.5 Hz, and the low-pass filter was set at 35 Hz LPN.

## 3. Results and Discussion

### 3.1. ME Fabrication and Characterization

The magnetic fluid is extruded from the mask-holes and forms the droplet array. Taking one magnetic droplet as the research object, when the magnetic droplet is placed in the magnetic field, the magnetic droplet is magnetized and drawn to form the microneedle due to the sum of gravity, surface tension and magnetic force. This is the magnetostatic Rosensweig surface. The change in potential energy due to the presence of the magnetized fluid is expressed as the sum of the gravitational, surface tension and magnetic potential energies as [27]:

(1) U=Ug+Ur+UM=12ρg∬​z2(x,y)dxdy+γ∬​1+(∇z)2dxdyU=Ug=+12[∭volumeμ0(H0)2dxdydz−∭fluidμ0MH0dxdydz]

in which, *H*_0_ is the value of vacuum magnetic field, *γ* the surface tension, *μ*_0_ the vacuum permeability, *ρ* the density and *M* the magnetic susceptibility of magnetic droplet. The equilibrium shape assumed by the magnetic fluid under these combined effects is such that the net energy of the system is minimized [28]. Thus, whereas a curved droplet surface having protrusions extending in the vertical direction increases the gravitational and surface tension potential energy, the elongated fluid features aligned with the magnetic field concomitantly decrease the magnetic potential energy until a balance is reached. As the sum of gravity, surface tension and magnetic force is zero and the net energy of the system is minimized, the microneedle shape is finally self-assembled. The self-assembled microneedle array in the magnetic field is heated and solidified due to polymerization reaction of epoxy resin. The fabricated MA also can be further applied to other fields, such as collection of micron-size oil droplets from water [29], transdermal drug delivery [30,31,32], biochemical sensor [33,34], cutaneous delivery of vaccine [35], fast drug detection [36], treatment of hydrocephalus [37], etc.

**Figure 6:**
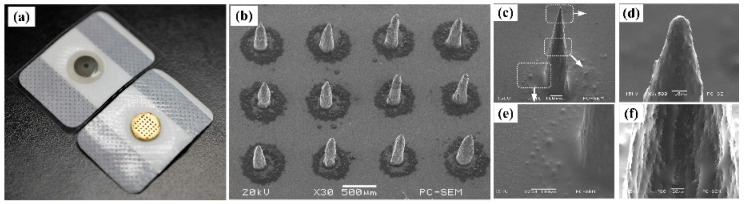
(**a**) ME and Ag/AgCl electrode; (**b**) SEM image of MA; (**c**) micro-needle; (**d**) micro-needle tip; (**e**) micro-needle bottom; and (**f**) micro-needle middle.

An image of the ME and Ag/AgCl electrode is shown in Figure 6a. The medical adhesive dressing could ensure the ME sticks on the human skin. The standard snap connector on the back of ME could guarantee the connection between ME and common bio-signal recording devices. There is a 6 × 6 magnetization-induced micro-needle array on the ME and the interval between adjacent micro-needles is 1 mm as shown in Figure 6b. The average length of the micro-needles is about 700 μm. The micro-needle diameter at the middle section is about 140 μm. The micro-needle looks like an inverted stalagmite as shown in Figure 6c.

The micro-needle tip looks like a cone and is sharp, as shown in Figure 6d. The tip radius is about 10 μm. The rough circle at the bottom around each micro-needle was formed as shown in Figure 6b,e. The surface of the micro-needle is rough due to the nano-iron powder surrounding it as shown in Figure 6f. The rough surface of the ME may increase the contact area and stability between ME and skin. Ti/Au films were coated on the whole surface of MA. The coating Ti film was used to increase the bonding strength between the MA and Au film [6]. The Au film coating can ensure the conductivity of the ME [6,15]. The epoxy resin is inert and limits adverse chemical reactions in the body. Furthermore, the whole surface of MA was coated with Ti/Au film. 316L stainless steel mask, Ti/Au films are all compatible, so the overall compatibility of the ME can be guaranteed.

### 3.2. Fracture Test

**Figure 7:**
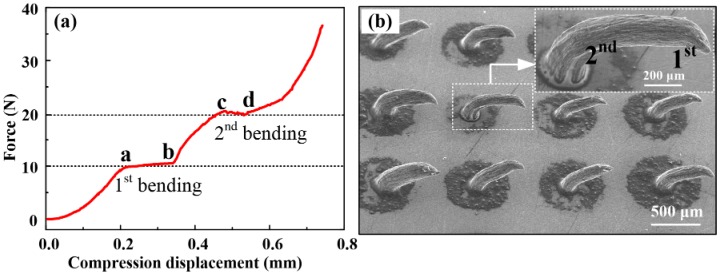
(**a**) Resistance force during the fracture test; and (**b**) SEM images of bent MEs after the fracture test.

Figure 7a presents the resistance force of the ME during the fracture test. Figure 7b is the SEM image of a bent ME after the fracture test. As the MA tips are in contact with the aluminum block, the resistance force linear increases with loading displacement. As the loading proceeds, micro-needles begin to yield. When the loading displacement is close to point “a”, the MA tips start to buckle and bend. The resistance force at the first bending is about 10 N. Thus, the maximum press force for the ME (buckling force) without being bent is about 10 N. The tips are seriously bent to about 90° at the point “b”. The first bends in tips are shown in Figure 7b. The resistance force increases rapidly as the MA is further compressed. This is a compression force along the axial direction of the micro-needles. As the compression force is beyond the buckling force, the micro-needles begin to bend again, this time at point “c”. The second bending at the middle section of the microneedle is also shown in Figure 7b. The resistance force at the second bending is about 20 N. The loading process is stopped when the resistance force is 45 N. The micro-needles could be bent without being broken as shown in Figure 7b. Above all, ME has strong toughness and strength which could prevent the micro-needles from breaking off in the skin during bio-signal recording.

### 3.3. Insertion Test ex Vivo

**Figure 8:**
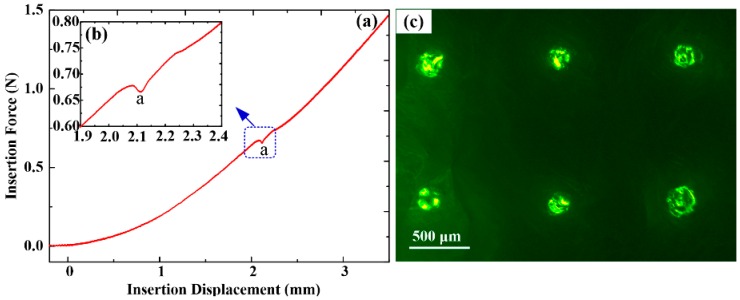
(**a**) Insertion force vs. displacement curve; (**b**) the penetration point; and (**c**) fluorescence image of punctured rabbit skin.

Figure 8a shows the insertion process of ME into rabbit skin ex vivo. The resistance force increases with the loading displacement once the MA tips contact the rabbit skin. The rabbit skin initially deforms under the advancing micro-needle tip. The inherent resistance property of skin which is regarded as the potential energy stored in the skin prevents MA penetration [38]. When the increasing pressure reaches the rupture limit of rabbit skin, the micro-needles penetrate into the skin at the point “a”. At this point, the stratum corneum is ruptured and the needles undergo a sudden movement into the skin. This movement is translated by the force sensor as a corresponding drop in the measured force as shown in Figure 8b. The minimum force required for micro-needle penetration into skin is defined as the penetration force [38,39]. The penetration force of MA into rabbit skin is about 0.68 N. This is comparable with the penetration force tested by Khanna et al. [38]. Suppose all micro-needles are punctured into skin and the penetration force for one micro-needle is 0.019 N. The penetration force of ME is at least an order smaller than the buckling force. It suggests that ME could easily penetrate into skin without being bent or buckled. At this critical force, the micro-needle tips crack and expand circumferential holes in the skin by stretching and tearing the skin, permitting the micro-needle tips to penetrate into skin. The skin puncture is a mode I crack [38]. The major potential energy is absorbed by the punctured skin. The resistance force increases slightly as the MA moves downwards. This is due to the resistance force converting compression force into sliding friction force between the micro-needles and skin. The punctured ME was observed by SEM and it was found that none of micro-needles were bent or broken due to their good rigidity and toughness. Figure 8c is a fluorescence image of the punctured rabbit skin. Several bright circular holes due to the microneedle diffusion of rhodamine B are neatly arranged, and the arrangement fits the micro-needle positions of ME. This indirectly suggests that the micro-needles of ME can insert in the skin without fracturing during the insertion process.

### 3.4. Bio-Signal Measurement

#### 3.4.1. EII Measurement

**Figure 9:**
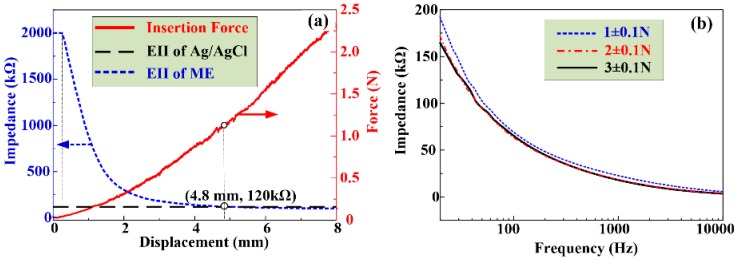
Insertion force and EII test. (**a**) EII during the insertion process; and (**b**) EII under different input voltage frequency.

Figure 9a shows the contact impedance between ME and forearm at the input voltage frequency of 50 Hz during the insertion process in vivo. The EII value is extremely large at the beginning and the insertion force is zero due to the absence of contact between the ME and human skin. Subsequently, the MA tips touch the forearm skin and the insertion force gradually increases. The EII value remains high due to the high impedance of the stratum corneum layer. As the compression force is about 17 mN, which is close to the penetration force for one micro-needle into rabbit skin (0.019 N), the EII drops suddenly. This may be due to the penetration of one micro-needle through the stratum corneum layer and a decrease of the EII between the ME and skin. As the insertion proceeds, the micro-needles of ME penetrate into the human skin one by one and the EII decreases with the insertion depth. It may be indirectly demonstrated by the small drops on the curve which is due to the large deformation of forearm skin during the insertion process. This insertion process in vivo is different from the insertion experiment ex vivo. When the compression force on the ME is larger than 1.2 N, the ME records a lower EII than that of Ag/AgCl electrodes (120 KΩ). This is attributed to the fact that the ME could puncture through the stratum corneum layer and eliminate the impedance influence of stratum corneum as shown in Figure 1. The stratum corneum is composed of high-densely packaged cornified cells which provide higher electrical impedance and attenuate signals [40]. When a force larger than 1.6 N is applied on the ME, the EII of the ME reaches a steady state, and its value is about 108 KΩ. The average force of an adult applying the ME with their thumb is about 20 N [41]. Therefore, we can easily press ME into human skin with a thumb and record bio-signals.

Figure 9b presents the ME skin-electrode impedances with different compression forces over the driving voltage frequency range from 20 Hz to 10 kHz. The EII of ME decreases with the frequency. The impedance of the human body is composed of resistance and capacitive resistance as shown in Figure 1. The capacitive resistance decreases with the frequency of input driving current. The EII of ME decreases with the insertion force at a given frequency [24]. The measured EII usually consists of electrode impedance, contact impedance and tissue impedance as shown in Figure 1. The contact impedance between the electrode and skin decreased with the insertion force since both the electrode impedance and tissue impedance are constant at a given voltage frequency. If the compression force is beyond 2 N, the impedance of ME varies little as shown in Figure 9a. It is further verified that the EII under compression 2 N and 3N are very close as shown in Figure 9b. Therefore, the ME can stably record EII or bio-signals beyond a compression force of 2 N which the subjects feel as almost painless.

#### 3.4.2. EMG Measurement

Figure 5 shows an EMG recording of the bicep brachii muscle. The EMG signals shown in Figure 5a,b were measured by Ag/AgCl electrodes and the ME, respectively. The signals measured by both the ME and Ag/AgCl electrodes fluctuate periodically along with the rhythmical contraction of the biceps brachii muscle. When the biceps brachii muscle is contracted from the relaxation state, the muscle cells are electrically or neurologically activated, and the electrical potential increases rapidly to a high amplitude. The signals acquired by both the ME and Ag/AgCl electrodes are similar in shape. This demonstrates that the ME could also accurately depict the EMG signals along with the activation of muscle in comparison with Ag/AgCl electrodes. The micro-needles of ME could penetrate through the stratum corneum and maintain good contact with viable epidermis. Therefore, ME is a good choice in EMG recording without skin preparation in comparison with Ag/AgCl electrodes.

#### 3.4.3. ECG Measurement

**Figure 10:**
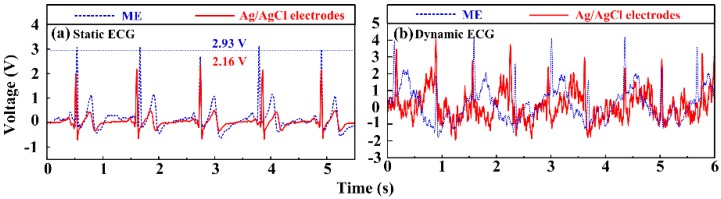
ECG signals recorded by Ag/AgCl electrodes and ME in the (**a**) static state; and (**b**) dynamic state.

Figure 10a presents a static ECG recording of Ag/AgCl electrodes and ME, respectively. The recording performances of ME are comparable with the Ag/AgCl electrode. The features of ECG signals, such as the QRS complex, T and P waves, are all distinguishable. The average peak values of R waves recorded by Ag/AgCl electrode and ME are 2.16 V and 2.93 V, respectively. The peak value of P waves and T waves measured by ME are also larger than those measured by the Ag/AgCl electrode, indicating that ME could collect bio-signals with lower attenuation. This may be attributed to the fact that that ME can pierce in the viable epidermis layer of skin and attenuate the impedance effect of the stratum corneum layer. Therefore, ME shows comparable or even better recording performance in comparison with Ag/AgCl electrode in static ECG tests. 

**Figure 11:**
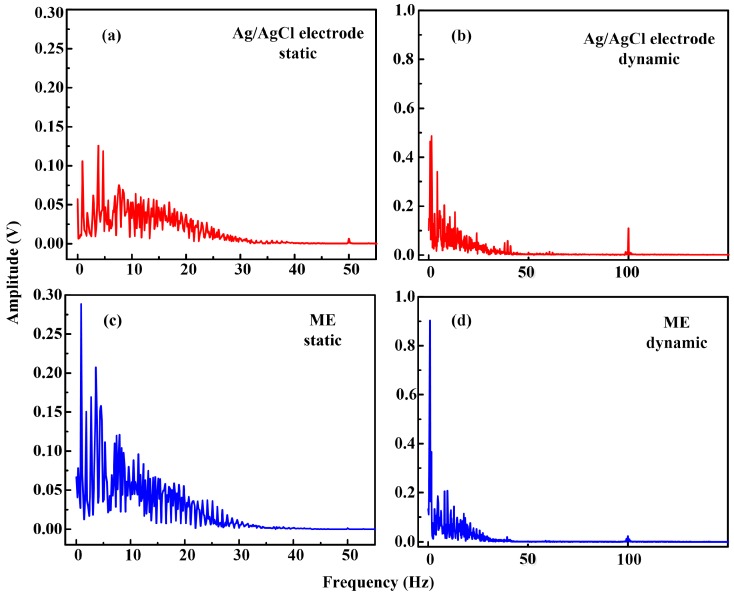
Frequency spectrum of ECG signals recorded by Ag/AgCl electrode and ME: (**a**,**c**) at the static state; and (**b**,**d**) at the dynamic state.

The signal quality of ECG measurement is seriously affected by motion artifacts [1,5,12]. Figure 10b presents a dynamic ECG recording of Ag/AgCl electrodes and the ME. The signal measured by the Ag/AgCl electrode is seriously disturbed by noise due to the motion artifacts. Only typical R waves are visible and the heart frequency rate can be figured out, while ECG signals collected by ME have more distinguishable QRS complexes, P waves and T waves than those by Ag/AgCl electrodes. As the arms swing during walking, the skin of the volunteers shifted, which modifies the relative Ag/AgCl electrode-skin position and causes the signal drifts. The microneedles of the ME can grasp the skin which prevents the relative position slip between skin and ME and maintains a relatively stable electrode-skin interface. Therefore, ME has potential to be a better choice for dynamic ECG signal recording. Figure 11 presents the frequency spectrum of ECG signals recorded by the Ag/AgCl electrodes and the ME. It shows similar amplitude profiles. However, the amplitude at low frequency recorded by the ME is larger. The frequency domain of the ECG signals varies from 0.05 to 100 Hz and is concentrated in the low frequency region. Moreover, the amplitude of the interference signal recorded by ME, especially at 50 Hz and 100 Hz, is lower. It suggests that ME is a good choice for ECG recording.

## 4. Conclusions

MEs were fabricated by the magnetization-induced self-assembly method for the purpose of bio-signal recording. The mechanical properties and bio-signal recording performance of the MEs were investigated by experiments. The main conclusions are:

(1) A MA can be self-assembled from a magnetic droplet array under the sum of gravitational, surface tension, and magnetic potential energies. The MEs were coated with Ti/Au films to guarantee their compatibility. The microneedle length is about 700 μm and its tip is sharp. Micro-needles of ME have good toughness and the buckling force is about 10 N. MEs also can easily pierce rabbit skin without being broken or buckling and their penetration force is about 0.68 N, so MEs can be easily fabricated by MSM and have good mechanical properties.
(2) The EII of a ME decreases rapidly as microneedles are pressed and pierced into the forearm skin one by one. The insertion process in vivo is different from that ex vivo due to the skin type and deformation. As the compression force pressed on the ME is larger than 2 N, the EII of ME reaches a steady constant value of about 108 KΩ which is lower than that measured by Ag/AgCl electrodes (120 KΩ), so a ME can stably record EII or bio-signals under a relative low compression force.
(3) The ME can depict the periodical fluctuations of EMG signals along with the rhythmical contraction of the biceps brachii muscle without skin preparation. The ME could record static ECG signals with a larger amplitude in comparison with a Ag/AgCl electrode due to the elimination of the stratum corneum layer impedance. Besides, the ME could collect more distinguishable dynamic ECG signals in comparison with the Ag/AgCl electrode, so the ME is a promising alternative electrode compared with conventional Ag/AgCl electrodes in some specific bio-signal recording situations.

## Figures and Tables

**Table 1:** Specific properties of epoxy Novolac resin and modified aliphatic amine.

Properties	Epoxy Novolac Resin	Aliphatic Amine	Unit
Density	1.22	1.05	g/mL
Viscosity	500 (66 °C)	50–110 (25 °C)	MPa·s
Chemical Formula	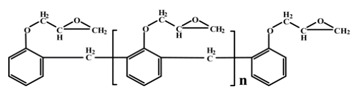	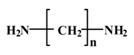	N/A
Polycondensation Equation	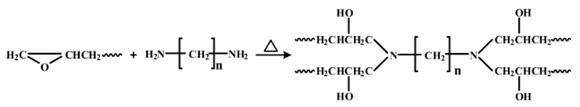		

**Table 2:** Specific parameters of iron powder.

Properties	Value	Unit
Diameter	50 ± 15	nm
Purity	>99.9%	N/A
Specific area	30	m^2^/g
Density	7.9	g/cm^3^
